# Silent Intruders: Air in the Cavernous Sinus and Neck Spaces–A Case Report

**DOI:** 10.2147/IMCRJ.S589643

**Published:** 2026-04-27

**Authors:** Muhammad Faisal Wadiwala, Anwar Ibrahim Joudeh, Sheik Akbar Hussain

**Affiliations:** 1 Department of Neurology, Al-Khor Hospital, Hamad Medical Corporation, Doha, 3050, Qatar; 2 Department of Internal Medicine, Al-Khor Hospital, Hamad Medical Corporation, Doha, 3050, Qatar; 3 Department of Medicine, Collage of Medicine, Qatar University, Doha, 2731, Qatar; 4 Department of Radiology, Al-Khor Hospital, Hamad Medical Corporation, Doha, 3050, Qatar

**Keywords:** air embolism, case report, cavernous sinus, cervical pneumatosis

## Abstract

**Introduction:**

Air in the cavernous sinus is rare and often linked to severe conditions like trauma, septic thrombosis, or gas-forming infections. However, advances in imaging and awareness of iatrogenic causes suggest it may also result from transient, self-limiting venous air embolism, especially after routine procedures like peripheral intravenous canulation.

**Case Presentation:**

A middle-aged diabetic male presented to the emergency department with dizziness and persistent nausea for two days. There was no history foe headache, fever or blurring of vision. On examination, he was severely dehydrated with reduced skin turgor and resting tachycardia. The patient was treated with intravenous hydration with improvement in his symptoms. Laboratory investigations showed only mild hyperglycemia with high lactic acid. Initial non-contrasted computed tomography (CT) of the head revealed air within the cavernous sinus and neck spaces. Four hours later, a repeated contrast-enhanced CT of the head and neck showed complete spontaneous resolution of the cavernous gas bubbles. Recent peripheral intravenous canulation was the likely cause of transient venous air embolism in this case.

**Conclusion:**

This case underscores the importance of differentiating benign, iatrogenic causes of pneumatosis from life-threatening conditions to prevent unnecessary interventions. Clinicians should consider recent intravenous canulation as a potential benign cause in clinically stable and asymptomatic patients, while systematically excluding other serious pathologies.

## Introduction

The presence of air within the cavernous sinus and neck spaces is a rare but clinically significant imaging finding, historically associated with severe pathologies such as skull base fractures, septic thrombosis, or gas-forming infections.^1^,^2^ The cavernous sinus, a critical neurovascular structure, is particularly susceptible to retrograde air migration due to its valveless venous connections and proximity to the skull base.^3^ Traditionally, the detection of air in this region warranted immediate concern for life-threatening conditions, necessitating aggressive diagnostic and therapeutic interventions. However, advancements in cross-sectional imaging and increased awareness of iatrogenic causes have revealed that such findings may also result from transient, self-limiting venous air embolism, particularly following routine medical procedures such as peripheral intravenous catheterization.^4^,^5^

This case underscores the presence of pneumatosis in an asymptomatic patient, challenging clinicians to distinguish benign iatrogenic causes from catastrophic pathologies. Accurate differentiation is crucial to avoid unnecessary interventions while ensuring life-threatening etiologies are not overlooked.

## Case Presentation

A 55-year-old Siri Lankan man presented to the emergency department with a 2-day history of dizziness and generalized weakness. He had severe nausea with decreased oral intake, but he did not have vomiting, abdominal pain, or change in his bowel motion. He also reported a dry cough for the past 15 days without any history of fever, chest pain, or shortness of breath. There was no history of headache, syncope, ear discharge, or tinnitus. His background history included type 2 diabetes for 8 years, for which he was only maintained on dietary and lifestyle modifications. He was not using any regular medications and did not have a history of previous surgery or hospitalization. He was not a smoker and did not consume alcohol beverages regularly. No history of illicit drug use.

On examination, the patient was initially dehydrated with dry mucous membranes and reduced skin turgor. His vital signs were the following: resting blood pressure 110/84 mmHg, pulse rate 118 beats per minute, respiratory rate 20 per minute, and his temperature was 36.9 C. The patient was conscious, alert, and oriented to time, place, and person. He was not pale or jaundiced. His respiratory, cardiovascular, and abdominal examinations were within the normal range. Neurological examination revealed no focal deficits, meningeal signs, or cranial nerve abnormalities.

**Figure 1:**
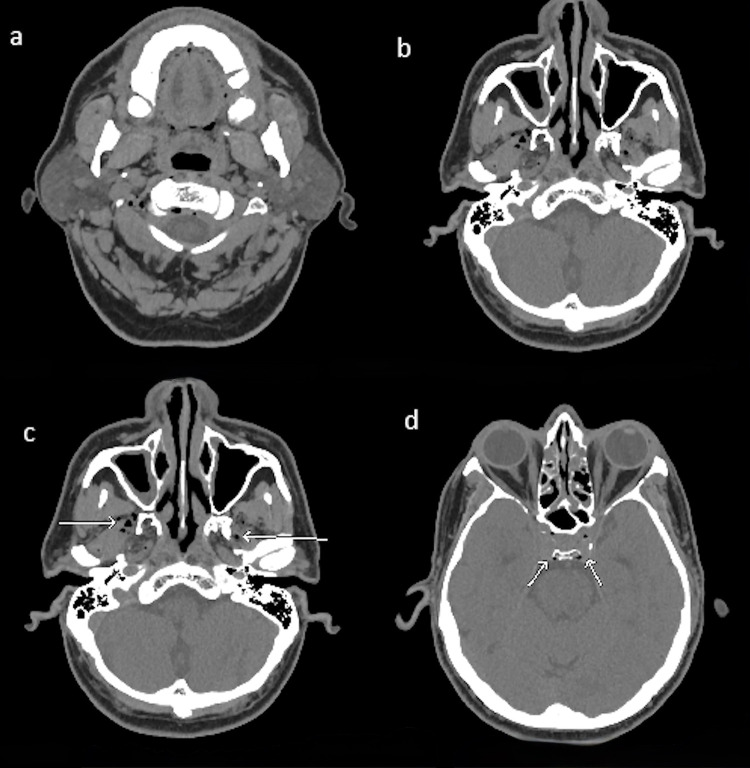
Initial non-contrasted computed tomography (CT) of the head and upper neck demonstrating venous air. (**a**) Axial section through the upper neck revealing small air foci in within the retro-dental soft tissues posterior to odontoid process, and the right paravertebral region venous plexus. (**b** and **c**) Axial images at the skull base showing air specks within the bilateral pterygoid venous plexuses (white arrow). (**d**) Axial bone window at the level of cavernous sinuses showing small air foci within both cavernous sinuses (white arrow).

A peripheral intravenous (IV) cannula was inserted to facilitate blood sample collection and the administration of intravenous fluids for hydration. His random blood glucose level was 10 mmol/L (Normal <7.8 mmol/L), and the remaining laboratory investigations, including a complete blood count and blood chemistry panel, were unremarkable except for an elevated lactic acid level of 8.0 mmol/L (normal range: 0.5–2.2 mmol/L). A chest X-ray was reported as normal. A non-contrasted computed tomography (CT) of the head revealed small air foci within the left cavernous sinus and pneumatosis along the venous sinuses as shown in Figure 1.

**Figure 2:**
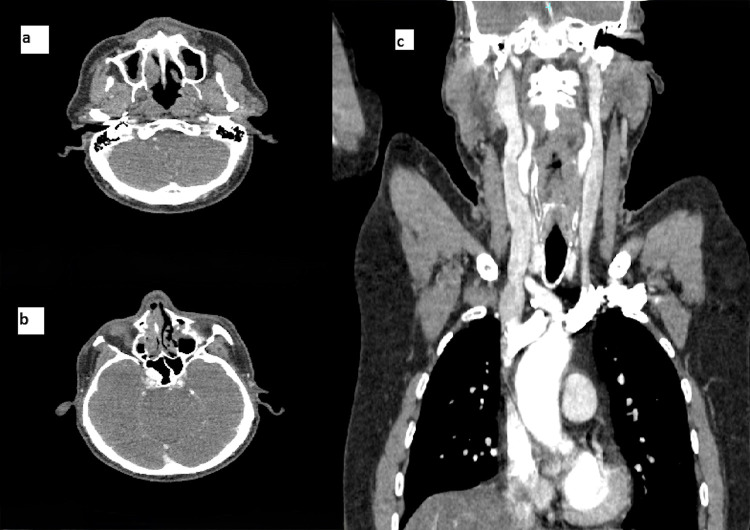
A follow-up contrast-enhanced CT scan. (**a**) Axial section showing the absence of air specks in the upper neck in the retrodental region and pterygoids. (**b**) Axial section showing normal enhancement of cavernous sinuses with no air specks. (**c**) Coronal contrasted neck CT showing patent internal jugular veins and normal vasculature with no sir specks in the soft tissues.

During his stay in the emergency department, the patient received three liters of isotonic saline, after which his symptoms improved. Approximately four hours later, a repeat contrast-enhanced head CT, along with CT scans of the neck and thorax, demonstrated complete resolution of the previously observed air specks in the cavernous sinus. The neck and thoracic structures appeared normal, with no evidence of extrapulmonary air as shown in Figure 2.

Given the complete resolution of symptoms and the disappearance of air within the cavernous sinus, the patient was diagnosed with transient air embolism, most likely secondary to peripheral cannulation. He was discharged home and remained asymptomatic on subsequent outpatient follow-up.

## Discussion

The presence of air in the cavernous sinus and neck spaces is a rare finding that warrants careful evaluation. Etiologies include benign iatrogenic causes such as venous gas embolism during IV access,^3^,^4^ traumatic causes necessitating exclusion of skull base fractures,^1^ or rare infection by gas-forming organisms.^2^

The pathophysiology of this case is centered on retrograde venous air migration during peripheral IV cannulation.^4^,^5^ The absence of valves in the jugular venous system allows air introduced into peripheral veins to travel against the venous flow, particularly in reclining patients, where buoyancy facilitates air movement superiorly.^6^,^7^ This mechanism aligns with prior reports of transient pneumocephalus following intravenous air injection, where air preferentially distributes to dependent intracranial structures such as the cavernous sinus.^4^,^8^

The rapid resolution of air in this patient within hours contrasts sharply with persistent air accumulation in infectious cases, where ongoing gas production sustains pneumatosis.^2^ Gas-forming infections such as sphenoid sinusitis often present with systemic signs of sepsis and progressive air accumulation,^2^ whereas iatrogenic venous air embolism typically resolves spontaneously.^4^,^5^

In contrast to this asymptomatic case, there are reports of symptomatic air in the cavernous sinus, often associated with more severe clinical presentations. For example, Kuno and Robertson described a patient with air in the cavernous sinus following orbital trauma, who presented with proptosis, ophthalmoplegia, and visual disturbances.^9^ Similarly, McCarthy et al reported a case of retrograde cerebral venous air embolism after central venous catheterization, where the patient experienced acute neurological deficits, including confusion and hemiparesis.^10^ These cases highlight the potential for air in the cavernous sinus to cause significant symptoms, particularly when associated with trauma or large volumes of air.

Recent literature highlights the evolving understanding of incidental venous air embolism.^4^,^5^ Earlier studies primarily associated it with central venous catheterization or surgical procedures, but contemporary reports increasingly document its occurrence during routine peripheral IV therapy.^4^,^5^ Conservative management suffices for asymptomatic patients, as small air volumes typically resorb without sequelae.^5^ Nevertheless, symptomatic cases, characterized by neurological deficits, chest pain, or cardiovascular instability, necessitate immediate interventions such as hyperbaric oxygen therapy to mitigate ischemic injury.^7^,^10^ Preventive strategies, including air-filter systems and meticulous catheter flushing techniques, may reduce the risk of iatrogenic embolism.^10^

In terms of imaging, non-contrast CT is frequently the initial imaging modality of choice due to its rapid acquisition, wide availability, and high sensitivity. Nevertheless, it might be limited by its relatively coarse spatial resolution and inability to differentiate intrasinus air from adjacent subarachnoid or epidural collections. In contrast, contrast-enhanced CT scan/CT angiography offers superior spatial resolution, enabling more precise delineation of the venous-cerebrospinal fluid interface, which facilitates accurate localization of air within the cavernous sinus. Additionally, it allows for simultaneous assessment of vascular structures, aiding in the exclusion of cavernous sinus thrombosis, stenosis, or anatomical variations. Consequently, contrast-enhanced CT/CT angiography provides incremental diagnostic value, particularly in cases where the diagnosis remains uncertain or clinical suspicion persists.

Notably, the presence of lactic acidosis alongside cerebral pneumatosis raised the concern for serious infections or other sinister pathologies. However, carful assessment of patient and thorough investigations did not show any evidence for sepsis, tissue ischemia or metabolic derangement. Additionally, the patient’s symptoms improved rapidly, and his vital signs stabilized after intravenous fluid resuscitation. Therefore, elevated lactate level was interpreted in the context of severe dehydration and relative hypoperfusion from prolonged poor oral intake rather than from an underlaying critical illness. Although cerebral pneumatosis is concerning on imaging, such findings frequently resolve spontaneously in asymptomatic patients, particularly in those with recent intravenous access.^4^ Clinicians must rigorously exclude infectious or thrombotic causes through history-taking, lab evaluation, and imaging.^1^,^2^

Despite the patient’s benign medical course, we acknowledge several limitations. The diagnosis of transient iatrogenic venous air embolism in this case was based on clinical stability and rapid radiologic resolution of intra-cavernous air without measuring air volume or confirming venous migration. In addition, we were not able to determine the exact timing and amount of air introduced during cannulation. However, we considered alternative diagnoses thoroughly. Cavernous sinus thrombosis was excluded due to the absence of fever, headache, cranial nerve deficits, orbital signs, or leukocytosis, and normal cavernous sinus opacification on follow-up imaging. Skull base fracture was excluded by the lack of trauma history and bony abnormalities on CT. Gas-forming infection was unlikely due to the patient’s afebrile state, normal inflammatory markers, imaging findings, and complete air resolution without antimicrobial therapy. Therefore, transient iatrogenic venous air embolism remains the most plausible explanation, but the diagnosis is ultimately one of exclusion.

This case emphasizes that air in the cavernous sinus and pneumatosis in the neck spaces are not necessarily serious findings. Recent medical procedures such as peripheral intravenous cannulation should be considered as a potential benign cause in clinically stable and asymptomatic patients. A thorough clinical correlation and short-term imaging follow-up can help differentiate transient iatrogenic air embolism from other sinister etiologies, thereby avoiding unnecessary invasive investigations or treatment.

## Data Availability

The data that support the findings of this study are not publicly available due to institutional privacy considerations but are available from the corresponding author upon reasonable request.
